# Actively or passively deacidified lysosomes push β-coronavirus egress

**DOI:** 10.1038/s41419-021-03501-5

**Published:** 2021-03-04

**Authors:** Xuefeng Wang, Gerry Melino, Yufang Shi

**Affiliations:** 1 The Third Affiliated Hospital of Soochow University and State Key Laboratory of Radiation Medicine and Protection, Institutes for Translational Medicine, Soochow University, 199 Renai Road, 215123 Suzhou, Jiangsu China; 2 CAS Key Laboratory of Tissue Microenvironment and Tumor, Shanghai Institute of Nutrition and Health, University of Chinese Academy of Sciences, Chinese Academy of Sciences, 320 Yueyang Road, 200031 Shanghai, China; 3 Department of Experimental Medicine, TOR, University of Rome Tor Vergata, Rome, 00133 Italy

**Keywords:** Mechanisms of disease, Lysosomes

In a very recent issue of Cell, Ghosh et al. describe how β-coronaviruses utilize deacidified lysosomes to egress from the infected cells and impair antigen presentation process^1^. This deacidified lysosome-mediated trafficking can be blocked by the Rab7 GTPase competitive inhibitor CID1067700 and alleviates β-coronavirus spread^1^.

SARS-Cov-2, one member of β-coronaviruses, brokes out all over the world in late 2019^2^. Although remdesivir, hydroxychloroquine, and other targeted drugs were tested in SARS-Cov-2 treatment, until now, there is no fully effective cure and vaccine^3–6^. What gives us a glimmer of hope is: not all people exposed to SARS-CoV-2 were infected and not all infected patients developed severe respiratory diseases^7,8^. This shows that the way the virus enters, replicates, and exits vary from person to person. A series of studies on SARS-Cov-2 have been reported, and the details of β-coronavirus entry and replication in the host cells have been understood, however, how newly assembled viruses egress from the host cells is still unclear. Now, Ghosh et al. made new and important contributions to help understand this unconventional egress process by describing that β-coronaviruses traffic to lysosomes and egress by Arl8b-dependent lysosomal exocytosis^1^. This non-lytic release happens with the lysosome deacidification, limited lysosomal degradation enzyme activation, and impaired antigen presentation^1^. This deacidified lysosome-dependent exocytosis may be the reason why it is difficult for the host to generate an efficient antivirus immune response and antibody production.

The interface between β-coronaviruses and host cells largely determines the infection and spread of the viruses. The egress pathway of β-coronaviruses has been assumed as a biosynthetic secretory vesicle-dependent plasma membrane trafficking manner^9^. The newly synthesized genomic RNA of β-coronaviruses is coated by virus N proteins and subsequently budding into ER–Golgi for the next process of egress trafficking. Nevertheless, Ghosh et al. verified the above hypothesis and found that is not the case. Brefeldin A (BFA), a small molecule for shutting down all anterograde biosynthetic secretory traffic from the ER/ERGIC out to the plasma membrane, has no effect on the egress of β-coronaviruses^1^. In addition, β-coronaviruses are enriched in the late endosomes and lysosomes during egress, and Rab7 inhibitor significantly inhibits β-coronavirus egress through interrupting biogenesis and maintenance of lysosomes. Therefore, β-coronavirus egress is dependent on a lysosomal manner, but not the previously assumed biosynthetic secretory pathway.

When a large number of β-coronaviruses are generated in the cells, the new viruses actively invade the lysosomes, or the lysosomes swallow the new viruses. Ghosh et al. found that plasma membrane LAMP1 levels have ~2.5-fold increase in the infected cells, indicating obvious fusion of lysosomes with the plasma membrane^1^. In addition, about two-fold increased cathepsin D and three-fold increased pro-cathepsin D secreted to the extracellular media of the infected cells, and the further research confirmed that lysosomes are deacidified and lysosomal enzymes are inactive in β-coronavirus-infected cells^1^. Given that acidified lysosomes are important for protease processing and antigen presentation of antigen-presenting cells (APCs), the β-coronavirus infection must be bound to affect the host’s immune response. This coincides with the clinical phenomenon that susceptible COVID-19 patients cannot utilize the immune system to effectively eliminate the SARS-Cov-2. Instead, excessive activation of non-antiviral immune cells can even cause autoimmune diseases^10^.

**Fig. 1:**
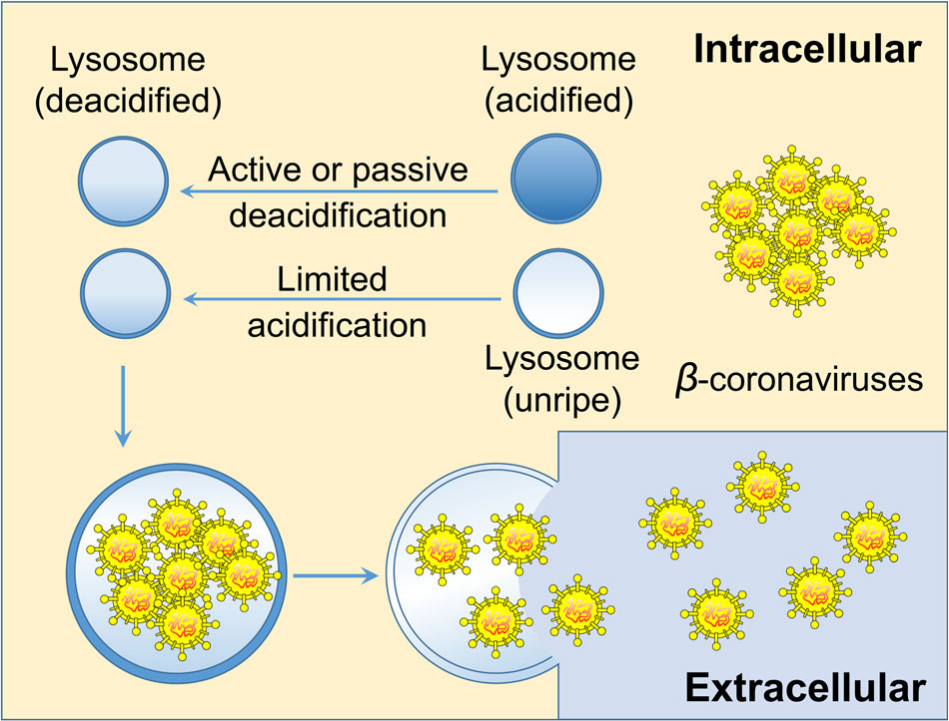
Actively or passively deacidified lysosomes push β-coronavirus egress. Lysosomal deacidification may be caused by β-coronavirus infection, or the infected cells actively deacidify lysosomes to release newly generated β-coronaviruses for relieving their own viral burden.

Coronaviruses have been detected in lysosomes at the late stages of infection for decades^11^. Ghosh et al. pioneered the study of the central role of lysosomes in the egress of β-coronaviruses, and CID1067700 limits egress of β-coronaviruses by reducing lysosomal number and inhibiting the maturation of endolysosomes. Although β-coronavirus egress depending on a lysosomal manner has been verified, it is still uncertain whether the lysosomal deacidification of cells is caused by a β-coronavirus infection, or whether the infected cells actively deacidify lysosomes to release newly generated β-coronaviruses for relieving their own viral pressure (Fig. 1). Some researchers think that lysosomal deacidification may be a consequence of the action of specific coronavirus proteins, and some researchers believe that lysosomal deacidification indirectly caused by too much cargo loading and/or the disturbed proton pump or ion channel trafficking^12,13^.

Recently, some studies have shown that lysosomal deacidification may be an active process of cells, for instance, insulin-like growth factor 2 receptor (IGF2R) activation promotes lysosomal deacidification-dependent proton rechanneling in maturing monocytes and dictated their oxidative phosphorylation metabolic inclination and anti-inflammatory potential^14^. Some interesting questions, provided that lysosomal deacidification is also an active process in cells infected by β-coronaviruses, what is the exact mechanism? Is it possible to artificially induce lysosome reacidification? Does re-acidifying of lysosomes limits β-coronavirus egress? As β-coronaviruses disrupted lysosome-dependent antigen cross-presentation pathways in the infected cells^1^. Simply inhibiting the lysosomal number and endolysosomal stability by Rab7-targeting inhibitors can only decrease β-coronavirus egress, however, the limited antigen presentation ability caused by impaired lysosomal acidification still cannot be restored. Therefore, it is necessary to explore the mechanism of lysosome deacidification and try to re-acidify lysosomes for inhibiting virus egress and restoring antigen presentation function of APCs. In addition, the identification of individual or clusters of predictive genetic alterations has been used for cancer predication^15^, therefore, examining the expression abundance of genes related to coronavirus emigration will be of great significance for distinguishing susceptible populations and formulating personalized prevention methods of COVID-19.

In summary, the β-coronavirus egress pathway involving lysosomal deacidification, impaired lysosomal degradation enzyme activation, and limited antigen presentation. This work highlight that targeting regulators of lysosomal trafficking and biogenesis, such as Arl8b and Rab7, may be the potential strategies to alleviate β-coronavirus infection and spread. More should be done in the future are to understand the mechanism of active and passive lysosome deacidification in β-coronavirus infected cells, to verify the effects of lysosomal reacidification on β-coronavirus egress and antigen presentation in APCs, and to extend the therapeutic potential of this discovery in the clinical treatment of β-coronavirus infection.
